# The use of Generative Artificial Intelligence in Scholarly Communication

**DOI:** 10.1590/1518-8345.0000.4560

**Published:** 2025-02-17

**Authors:** Lilian Nassi-Calò

**Affiliations:** 1 Associate Editor of the Pan American Journal of Public Health of the PAHO/WHO and SciELO Program Consultant, São Paulo, SP, Brazil

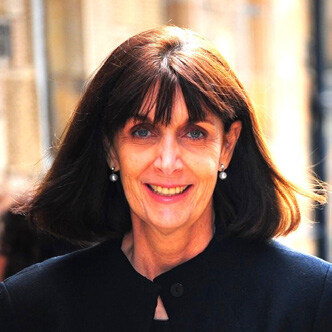



In the second decade of the 21^st^ century, the academic community witnessed the emergence of Generative Artificial Intelligence (AI)^(1)^ and Large Language Models^(2)^ (LLM), as well as tools such as ChatGPT, Gemini, Copilot and others that became part of everyday conversations and discussions about their potential use in research and scholarly communication, perhaps signaling the prelude to the third digital transformation of scholarly publishing^(3)^.

AI was legitimized by the highest forum of academic merit with a double award in 2024. The first was the Nobel Prize in Physics for J. Hopfield and G. Hinton^(4)^ for their fundamental discoveries and inventions that make machine learning possible with artificial neural networks, the basis for generative models of artificial intelligence, which allow machines not only to speak, draw and create music like us, but to continue learning how to do all this better and better. The second was the Nobel Prize in Chemistry awarded to D. Baker, D. Hassabis and J. Jumper^(5)^ for their work on predicting the structure of proteins using AI – something that has challenged scientists for decades – and for the possibility of creating hypothetical proteins with numerous therapeutic applications.

The awards, point out The New York Times^(6)^, indicate a paradigm shift in science from the traditional way in which the Nobel Committee selected recipients to receive the honor. When awarding an AI contribution, such as the Nobel in Chemistry, for example, it would also be necessary to recognize the researchers with whose results the AI has been trained, which can amount to hundreds or thousands of contributions.

With regard to AI-generated texts in scientific articles, at first many journal editors were looking for software^(7-11)^ to detect such texts in order to reject manuscripts that had been partially or completely written with such tools.

Today, the trend, according to the scientific associations and publishers consulted^(12-20)^, is to consider publishing texts that have been revised, translated, edited, corrected or written with the help of a chatbot, as long as the chatbot is not listed as the author or co-author, since AI does not comply with the ICMJE authorship criteria^(13)^. Furthermore, authors must declare the use of chatbots in the methods section, specifying the prompts used; and be aware that they are responsible for all material generated by the AI and for properly attributing (citing) all sources. It is therefore the author’s responsibility to ensure that the content generated by AI reflects their data and ideas and does not contain plagiarism, fabricated data or any falsification^(20)^. It is important to note that all materials fed into AI tools and chatbots become learning material for this AI and therefore lose its unpublished character. For this reason, it is not recommended to use generative AI to detect forms of plagiarism, copyright infringement and other forms of misconduct, although its use can be extended to other activities in the context of open science^(21-22)^.

As well put by C. Leonard^(20)^, in a recent post on The Scholarly Kitchen blog, “*Is AI the Answer to Peer Review Problems, or the Problem Itself?*”, the answer, unfortunately, is that AI will not be the solution to peer review. Its use may seem helpful, but it is a task that always requires human supervision.

**Figure 1 -:**
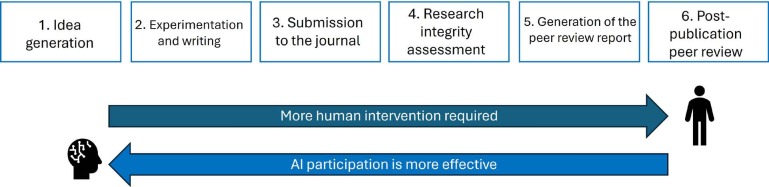
Stages of the scientific enterprise in which human and AI participation, respectively, have the greatest chance of success (Adapted from Leonard, C. 2024)^(23)^

The diagram in Figure 1 shows how humans and AI, respectively, are more likely to succeed in the stages of the scientific process.

A peer review report generated by LLM may seem reasonable: it contains a summary of the article, some of its strengths and weaknesses, areas for improvement and a general recommendation on acceptance, revision to a greater or lesser degree, or rejection. However, there is little or no comparison to literature in the field (something the human reviewer would be able to do), and no assessment of the innovative nature of the article. In addition, the suggested references are prone to “hallucinations” and there is a tendency to evaluate all manuscripts with “the article needs a little revision”. However, always considering the potential breach of confidentiality, AI could carry out a preliminary review, to be completed by human reviewers, diversifying the pool of referees and reducing bias. An interesting SWOT analysis of the roles of AI and human reviewers in peer review provides additional considerations^(24)^. It is worth mentioning that most publishers and scientific associations advise against and even prohibit the use of AI in peer review.

And to find out what scholars think about AI and science, a survey^(25)^ was carried out with 1,600 scientists in September 2023. As positive aspects, respondents mentioned that AI “provides faster ways to process data”; “speeds up computing” and “saves time and financial resources”, among others. As negative aspects, they mentioned that “it leads to greater reliance on pattern recognition without understanding”; “the results can highlight bias or discrimination in the data”; “it facilitates fraud” and “inappropriate use leads to irreproducible research”, among others. Regarding the use of LLMs (such as ChatGPT), the researchers mentioned “support in writing software code”; “brainstorming research ideas”, and “support in scientific writing”. As for the quality of the peer review carried out by AI, only 16% of researchers consider it to be adequate.

Interestingly, many mentioned the fear that the continued use of AI would produce and disseminate false information. They were unanimous, however, in stating that AI and LLM are “*here to stay*”. One researcher added to the comment, “*we have to focus now on how to ensure that it brings us more benefits than problems*”. This reflection, I believe, sums up the expectation of the scientific community and society about AI-based developments.
