# Supplementary material for: The use of Generative Artificial Intelligence in Scholarly Communication
Source: Rev Lat Am Enfermagem. 2025 Feb 17;33:e4560. doi: 10.1590/1518-8345.0000.4560 (PMC11835001; doi:10.1590/1518-8345.0000.4560)
Supplement: Translation

# Translation: O uso da Inteligência Artificial Generativa na Comunicação Científica

**DOI:** 10.1590/1518-8345.0000.4561

**Authors:** Lilian Nassi-Calò

**Affiliations:** 1 Editora Associada da Revista Panamericana de Salud Pública da OPAS/OMS e consultora do Programa SciELO, São Paulo, SP, Brasil.



Na segunda década do século XXI, a comunidade acadêmica assistiu ao surgimento da Inteligência Artificial Generativa (IA)^(1)^ e de *Large Language Models* ^(2)^ (LLM), além de ferramentas como o *ChatGPT*, *Gemini*, *Copilot* e outras que passaram a fazer parte das conversas quotidianas e discussões sobre seu potencial uso na pesquisa e na comunicação científica sinalizando, talvez, o prelúdio da terceira transformação digital da publicação acadêmica^(3)^.

A legitimação da IA pelo mais elevado fórum do mérito acadêmico se deu com uma dupla premiação, em 2024. A primeira, com o Nobel de Física a J. Hopfield e G. Hinton^(4)^ por suas descobertas e invenções fundamentais que possibilitam o aprendizado de máquina com redes neurais artificiais, a base para modelos generativos de inteligência artificial, que faz com que as máquinas não apenas falem, desenhem e criem música como nós, mas sigam aprendendo a como fazer tudo isso cada vez melhor. A segunda, com o Nobel de Química a D. Baker, D. Hassabis e J. Jumper^(5)^ por seu trabalho sobre a previsão de estrutura de proteínas por IA – algo que desafia os cientistas há décadas – e pela possibilidade de criar proteínas hipotéticas, com inúmeras aplicações terapêuticas.

As distinções, ressalta o New York Times^(6)^, indicam uma mudança de paradigma na ciência na forma tradicional com a qual o Comitê Nobel selecionava os recipientes para receber a honraria. Ao premiar uma contribuição da IA, como o Nobel de Química, por exemplo, seria necessário reconhecer também os pesquisadores com cujos resultados a IA foi treinada, que pode atingir centenas ou milhares de contribuições.

No que concerne a textos gerados por IA em artigos científicos, a princípio, muitos editores de periódicos buscavam *software* ^(7-11)^ para detectar tais textos, de forma a rejeitar manuscritos parcial ou totalmente redigidos com tais ferramentas.

Hoje, a tendência, segundo as associações científicas e *publishers* consultados^(12-20)^, é considerar a publicação de textos revisados, traduzidos, editados, corrigidos ou escritos com a ajuda de um *chatbot*, desde que este não seja listado como autor ou coautor, uma vez que a IA não cumpre com os critérios de autoria do ICMJE^(13)^. Ademais, os autores devem declarar o uso dos *chatbots* na seção de métodos, especificando os *prompts* utilizados; e estar cientes de que são responsáveis por todo o material gerado pela IA e pela atribuição (citação) apropriada de todas as fontes. É, portanto, responsabilidade do autor assegurar que o conteúdo gerado pela IA reflita seus dados e suas ideias e não contenha plágio, dados fabricados ou qualquer falsificação^(20)^. É importante salientar que todo material alimentado em ferramentas de AI e *chatbots* se torna material de aprendizado desta IA, e perde, portanto, seu caráter inédito. Por esta razão, não é recomendável utilizar IA generativa para detectar formas de plágio, violação de direitos autorais e outras formas de má conduta, embora seu uso possa se estender a outras atividades no contexto da ciência aberta^(21-22)^.

Como bem colocado por C. Leonard^(20)^, em um recente post do blog *The Scholarly Kitchen*, “*Is AI the Answer to Peer Review Problems, or the Problem Itself?*”, a resposta, infelizmente, é que a IA não será a solução para a avaliação por pares. Seu uso pode até parecer útil, porém é uma tarefa que não prescinde da supervisão humana.

O diagrama da Figura 1 mostra como seres humanos e IA, respectivamente, têm maior propensão a ser bem-sucedidos nas etapas do processo científico.

**Figura 1 -:** Etapas do empreendimento científico nas quais a participação humana e da IA, respectivamente, têm maior possibilidade de sucesso (Adaptado de Leonard, C. 2024)^(23)^

Um informe de avaliação gerado por LLM pode parecer razoável: contém um resumo do artigo, algumas de suas fortalezas e debilidades, áreas para aperfeiçoamento e uma recomendação geral sobre aceitação, revisão em maior ou menor grau ou rejeição. No entanto, há pouca ou nenhuma comparação com a literatura da área (algo que o parecerista humano estaria apto a realizar), e não há avaliação do caráter inovador do artigo. Ademais, as referências sugeridas são propensas a “alucinações” e há uma tendência de avaliar todos os manuscritos com “o artigo necessita de uma pequena revisão”. No entanto, sempre considerando a potencial quebra de confidencialidade, a IA poderia realizar uma revisão preliminar, a ser completada por pareceristas humanos, diversificando o *pool* de revisores e reduzindo o viés. Uma interessante análise SWOT das atribuições da IA e de revisores humanos na avaliação por pares traz considerações adicionais^(24)^. Cabe mencionar que a maior parte dos *publishers* e associações científicas desaconselha e até proíbe o uso da IA na avaliação por pares.

E para saber o que pensam os acadêmicos sobre IA e ciência, foi realizada uma pesquisa^(25)^ com 1.600 cientistas, em setembro de 2023. Como aspectos positivos os respondentes mencionaram que a IA “provê formas mais rápidas de processar dados”; “acelera a computação” e “economiza tempo e recursos financeiros”, entre outros. Como aspectos negativos, mencionaram que “leva a maior dependência do reconhecimento de padrões sem compreensão”; “os resultados podem salientar preconceitos ou discriminação nos dados”; “facilita a fraude” e “o uso inadequado leva a pesquisas irreprodutíveis”, entre outros. Quanto ao uso de LLMs (como o *ChatGPT*), os pesquisadores mencionaram “apoio na escrita de códigos de software”; “*brainstorm* de ideias de pesquisa”, e “apoio na escrita científica. Quanto à qualidade da avaliação por pares realizada pela IA, apenas 16% dos pesquisadores a consideram adequada.

É interessante observar que muitos mencionaram o temor de o uso continuado da IA produzir e disseminar informação falsa. Foram unânimes, entretanto, em afirmar que a IA e LLM estão “aqui para ficar”. Um pesquisador adicionou ao comentário, “temos que nos concentrar agora em como garantir que nos traga mais benefícios do que problemas”. Esta reflexão, acredito, resume a expectativa da comunidade científica e da sociedade sobre os desenvolvimentos baseados em IA.

# Translation: El uso de la Inteligencia Artificial Generativa en la Comunicación Científica

**DOI:** 10.1590/1518-8345.0000.4559

**Authors:** Lilian Nassi-Calò

**Affiliations:** 1 Editora Asociada da Revista Panamericana de Salud Pública da OPS/OMS e consultora del Programa SciELO, São Paulo, SP, Brasil.



En la segunda década del siglo XXI, la comunidad académica fue testigo de la aparición de la Inteligencia Artificial Generativa (IA)^(1)^ y los *Large Language Models* ^(2)^ (LLM), así como de herramientas como *ChatGPT*, *Gemini*, *Copilot* y otras que pasaron a formar parte de las conversaciones y discusiones cotidianas sobre su potencial uso en la investigación y la comunicación científica, señalando quizás el preludio de la tercera transformación digital de la publicación científica^(3)^.

La IA fue legitimada por el máximo foro del mérito académico con un doble galardón, en 2024. En primer lugar, se concedió el Premio Nobel de Física a J. Hopfield y G. Hinton^(4)^ por sus descubrimientos e inventos fundamentales que hacen posible el aprendizaje automático con redes neuronales artificiales, base de los modelos generativos de inteligencia artificial, lo que significa que las máquinas no sólo hablan, dibujan y crean música como nosotros, sino que siguen aprendiendo a hacer todo esto cada vez mejor. El segundo fue el Premio Nobel de Química concedido a D. Baker, D. Hassabis y J. Jumper^(5)^ por sus trabajos sobre la predicción de la estructura de las proteínas mediante IA – algo que ha supuesto un reto para los científicos durante décadas – y por la posibilidad de crear proteínas hipotéticas con numerosas aplicaciones terapéuticas.

Los premios, señala el New York Times^(6)^, indican un cambio de paradigma en la ciencia respecto a la forma tradicional en que el Comité Nobel seleccionaba a los galardonados para recibir el honor. Al premiar una contribución de la IA, como el Nobel de Química, por ejemplo, sería necesario reconocer también a los investigadores con cuyos resultados se ha entrenado la IA, lo que puede ascender a cientos o miles de contribuciones.

En lo que respecta a los textos generados por IA en artículos científicos, al principio muchos editores de revistas buscaban *software* ^(7-11)^ para detectar este tipo de textos con el fin de rechazar manuscritos que hubieran sido parcial o totalmente escritos con este tipo de herramientas.

Hoy en día, la tendencia, según las asociaciones científicas y *publishers* consultados^(12-20)^, es considerar la publicación de textos que hayan sido revisados, traducidos, editados, corregidos o escritos con la ayuda de un chatbot, siempre y cuando el chatbot no figure como autor o coautor, ya que la IA no cumple los criterios de autoría del ICMJE^(13)^. Además, los autores deben declarar el uso de los *chatbots* en la sección de métodos, especificando los *prompts* utilizados; y ser conscientes de que son responsables de todo el material generado por la IA y de atribuir (citar) correctamente todas las fuentes. Por lo tanto, es responsabilidad del autor asegurarse de que el contenido generado por la IA refleja sus datos e ideas y no contiene plagios, datos inventados o falsificaciones^(20)^. Es importante destacar que todo el material introducido en herramientas de IA y *chatbots* se convierte en material de aprendizaje para esta IA, por lo que pierde su carácter inédito. Por esta razón, no se recomienda el uso de IA generativa para detectar formas de plagio, infracción de derechos de autor y otras formas de mala conducta, aunque su uso puede extenderse a otras actividades en el contexto de la ciencia abierta^(21-22)^.

Como bien expone C. Leonard^(20)^, en un reciente post del blog *The Scholarly Kitchen*, “*Is AI the Answer to Peer Review Problems, or the Problem Itself?*”, la respuesta, desafortunadamente, es que la IA no será la solución a la revisión por pares. Su uso puede parecer útil, pero es una tarea que siempre requiere supervisión humana.

El diagrama de la Figura 1 muestra cómo los humanos y la IA, respectivamente, tienen más probabilidades de éxito en las etapas del proceso científico.

**Figura 1 -:** Etapas del emprendimiento científico en las que la participación humana y la IA, respectivamente, tienen más posibilidades de éxito (Adaptado de Leonard, C. 2024)^(23)^

Una revisión generada por LLM puede parecer razonable: contiene un resumen del artículo, algunos de sus puntos fuertes y débiles, áreas de mejora y una recomendación general sobre su aceptación; revisión en mayor o menor grado o rechazo. Sin embargo, hay poca o ninguna comparación con la literatura en el campo (algo que el revisor humano sería capaz de hacer), y ninguna evaluación de la naturaleza innovadora del artículo. Además, las referencias sugeridas son propensas a las “alucinaciones” y se tiende a evaluar todos los manuscritos con un “el artículo necesita una pequeña revisión”. Sin embargo, siempre teniendo en cuenta la posible violación de confidencialidad, la IA podría llevar a cabo una revisión preliminar, que completarían árbitros humanos, diversificando el grupo de revisores y reduciendo el sesgo. Un interesante análisis DAFO de las funciones de la IA y los revisores humanos en la revisión por pares aporta consideraciones adicionales^(24)^. Cabe mencionar que la mayoría de los *publishers* y asociaciones científicas desaconsejan e incluso prohíben el uso de la IA en la revisión por pares.

Y para conocer la opinión de los académicos sobre la IA y la ciencia, en septiembre de 2023 se realizó una encuesta^(25)^ a 1.600 científicos. Como aspectos positivos, los encuestados mencionaron que la IA “proporciona formas más rápidas de procesar datos”; “acelera la computación” y “ahorra tiempo y recursos financieros”, entre otros. Como aspectos negativos, mencionaron que “conduce a una mayor dependencia de reconocer patrones sin comprenderlos”; “los resultados pueden poner de manifiesto sesgos o discriminación en los datos”; “facilita el fraude” y “un uso inadecuado conduce a investigaciones irreproducibles”, entre otros. En cuanto al uso de LLM (como *ChatGPT*), los investigadores mencionaron “apoyo en la escritura de código de software”; “lluvia de ideas de investigación” y “apoyo en la escritura científica”. En cuanto a la calidad de la revisión por pares llevada a cabo por la IA, sólo el 16% de los investigadores la considera adecuada.

Es interesante señalar que muchos mencionaron el temor a que el uso continuado de la IA produjera y difundiera información falsa. Sin embargo, fueron unánimes al afirmar que la IA y el LLM “están aquí para quedarse”. Un investigador añadió al comentario: “tenemos que centrarnos en cómo garantizar que nos aporte más beneficios que problemas”. Creo que esta reflexión resume las expectativas de la comunidad científica y de la sociedad sobre los avances basados en la IA.
